# Combination of ICP-MS, capillary electrophoresis, and their hyphenation for probing Ru(III) metallodrug–DNA interactions

**DOI:** 10.1007/s00216-017-0186-0

**Published:** 2017-01-23

**Authors:** Lidia S. Foteeva, Magdalena Matczuk, Katarzyna Pawlak, Svetlana S. Aleksenko, Sergey V. Nosenko, Vasily K. Karandashev, Maciej Jarosz, Andrei R. Timerbaev

**Affiliations:** 1 Vernadsky Institute of Geochemistry and Analytical Chemistry, Kosygin St. 19, 119991 Moscow, Russian Federation; 2 Chair of Analytical Chemistry, Faculty of Chemistry, Warsaw University of Technology, Noakowskiego St. 3, 00-664 Warsaw, Poland; 3 Saratov State University, Astrakhanskaya St. 83, 410012 Saratov, Russian Federation; 4 Institute of Microelectronics Technology and High-Purity Materials, Acad. Ossipyan St. 6, 142432 Chernologolovka, Russian Federation

**Keywords:** Anticancer metallodrugs, DNA, ICP-MS, Capillary electrophoresis

## Abstract

Determination of the DNA-binding reactivity and affinity is an important part of a successful program for the selection of metallodrug candidates. For such assaying, a range of complementary analytical techniques was proposed and tested here using one of few anticancer metal-based drugs that are currently in clinical trials, indazolium *trans-*[tetrachloridobis(1*H*-indazole)ruthenate(III), and a DNA oligonucleotide. A high reactivity of the Ru drug was confirmed in affinity capillary electrophoresis (CE) mode, where adduct formation takes place in situ (i.e., in the capillary filled with an oligonucleotide-containing electrolyte). To further characterize the binding kinetics, a drug–oligonucleotide mixture was incubated for a different period of time, followed by ultrafiltration separation into two different in molecular weight fractions (>3 and <3 kDa). The time-dependent distribution profiles of the Ru drug were then assessed by CE-inductively coupled plasma mass spectrometry (ICP-MS), revealing that at least two DNA adducts exist at equilibrium conditions. Using standalone ICP-MS, dominant equilibrium amount of the bound ruthenium was found to occur in a fraction of 5–10 kDa, which includes the oligonucleotide (ca. 6 kDa). Importantly, in all three assays, the drug was used for the first time in in-vitro studies, not in the intact form but as its active species released from the transferrin adduct at simulated cancer cytosolic conditions. This circumstance makes the established analytical platform promising to provide a detailed view on metallodrug targeting, including other possible biomolecules and ex vivo samples.

**Electronic supplementary material:**

The online version of this article (doi:10.1007/s00216-017-0186-0) contains supplementary material, which is available to authorized users.

## Introduction

Anticancer metallodrugs seem to have a great many targets such as DNA, proteins, membranes, etc. However, the true lesion responsible for the biological activity of a drug is difficult to determine. This is not only because metal complexes are naturally diversely reactive species. There are at least two additional obstacles. First, analytical techniques and tools being used to map interactions with different pertinent biomolecules are not always of the metallomic origin and, as such, incapable to identify, characterize, and quantify metal species stemmed from binding. Then the path taken by a metal complex in reaching its target and accompanying changes in metal speciation are typically downplayed.

In an attempt to resolve these challenges, the present study was designed around an experimental anticancer ruthenium-based drug, indazolium *trans-*[tetrachloridobis(1*H*-indazole)ruthenate(III), and DNA, representing a fruitful target for metal complexes [1–3]. The selection of this metallodrug was governed not only by the advanced status of its development [4, 5] but also by a great deal of accumulated knowledge regarding the chemistry behind a drug’s delivery, uptake, and cell processing, including activation and targeting [6–9]. While DNA is thought to be not the main target for this (as well as any other) Ru drug [10, 11], no other cell component is unambiguously proven to play this role. To simplify the metal–bio system under scrutiny, a DNA oligonucleotide rich of GMP, the major nucleotide binding metallodrugs (including the ruthenium drug of interest [12, 13]), was employed in binding experiments (as a DNA sequence simpler than the more heterogeneous genomic DNA).

In a basic metallomic approach, a highly sensitive metal-specific method, inductively coupled plasma mass spectrometry (ICP-MS), is used in on-line combination with a high-throughput separation technique. Capillary electrophoresis (CE) constitutes a powerful method to resolve mixtures of metallodrugs, their metabolites, and adducts with biomolecules, including DNA binding blocks (such as GMP [12, 13]), prior to ICP-MS detection [14–16]. In situations where more accurate binding information is requested, ICP-MS quantification following fractionation of a drug–biomolecule mixture by ultrafiltration is preferable [17] (though at the expense of on-line configuration). Whichever individual metallomic (or complementary) technique is in use, their combined application would help gain a deeper understanding of biotransformations for a given complex. This strategy was adopted here to develop a versatile analytical platform for characterization of different metallodrug–DNA systems.

## Materials and methods

### Materials

The lyophilized powder of DNA oligonucleotide (5'- GTC GTA CTG ATA CAT GAG CC –'3; 6117 Da) was the product of Genomed (Warsaw, Poland) or Syntol (Moscow, Russia). It was used as an aqueous solution prepared in accordance with the producer recommendations and stored at –20 °C no longer than 1 month. Indazolium *trans*-[tetrachloridobis(1*H*-indazole)ruthenate(III)] was synthesized at the University of Vienna (see the Electronic Supplementary Material (ESM) for chemical formula). The stock solution of the Ru drug (1 mM) was prepared daily in 100 mM NaCl. Human transferrin (>98%), glutathione (>98%), and L-ascorbic acid (>99.5%) were purchased from Sigma-Aldrich (St. Louis, USA). Sodium chloride, sodium dihydrogen phosphate, disodium hydrogen phosphate, also from Sigma-Aldrich (St. Louis, USA), were of analytical reagent grade. Sodium hydroxide and citric acid (>99.5%) were obtained from Fluka (Buchs, Switzerland). High-purity water (18 MΩ cm^–1^) used throughout this work was obtained from a Milli-Q water purification system (Millipore Elix 3; Saint-Quentin, France).

### Instrumentation

A X-7 Thermo Elemental and an Agilent 7500a mass spectrometers were used for off-line and hyphenated ICP-MS measurements, respectively. In case of standalone operation, the instrument was equipped with a standard low-volume glass spray chamber (Peltier, cooled at 3 °C), and a concentric glass PolyCon nebulizer operating at a sample uptake rate of 0.8 mL min^–1^. Before analysis, the instrument was tuned to achieve maximum sensitivity and ^115^In served as internal standard. For coupling to CE, a torch with a smaller inlet (1.5 mm) was utilized to minimize the influence of plasma backpressure on the electrophoretic flow, and a platinum shield was installed into the torch to improve sensitivity. Fitted with a microconcentric nebulizer CEI-100 (CETAC, Omaha, NE, USA), the mass spectrometer was interfaced with an Agilent HP^3D^ CE system (Waldbronn, Germany). Affinity CE was performed using a CAPEL 105 (Lumex, St. Petersburg, Russia).

Fused-silica capillaries of inner diameter of 75 *μ*m and total length as specified below were obtained from CM Scientific Ltd. (Silsden, UK) or BGB Analytik (Schlossboeckelheim, Germany). Prior to the first use, the capillary was flushed at 1 bar with 1 M NaOH and water (30 min each). The capillary cassette and sample tray were thermostatted at 37 °C. Samples were introduced into the capillary hydrodynamically. A high voltage with a positive polarity placed at the inlet end of the capillary was applied to generate separations. Instrument control and data analysis were performed using ChemStation (Agilent) or Elforan (Lumex) software. The main instrumental and operational parameters are presented in Table 1.

**Table 1:** Operational parameters and settings

ICP-MS	Standalone	Hyphenated
Plasma gas flow rate	12 L min^–1^	15 L min^–1^
Auxiliary gas flow rate	0.9 L min^–1^	0.9 L min^–1^
Plasma rf power	1250 W	1290 W
Isotopes monitored	^101^Ru, ^102^Ru, ^115^In	^102^Ru, ^57^Fe, ^72^Ge
Dwell time	1 ms	100 ms
Interface		
Spray chamber volume	5 mL	
Nebulizer gas flow rate	1.0 L min^–1^	
CE		
Capillary	Fused-silica, inner diameter 75 *μ*m, length 70 cm	
BGE	10 mM NaH_2_PO_4_–10 mM Na_2_HPO_4_, 4 mM NaCl, pH 6.0	
Sample introduction	30 mbar for 10 s (injection volume 25.5 nL)	
Voltage	25 kV	
Current	28–32 *μ*A	
Sheath liquid	1 mM phosphate buffer (pH 6.0), 0.4 mM NaCl, 20 *μ*g L^–1^ Ge	
Affinity CE		
Capillary	Fused-silica, inner diameter 75 *μ*m, length 60/50.5 cm	
BGE	10 mM NaH_2_PO_4_–10 mM Na_2_HPO_4_, 4 mM NaCl, pH 6.0, 2 × 10^–5^ M oligonucleotide	
Sample introduction	10 mbar for 5 s (4 nL)	
Voltage	10 kV	
Current	47 *μ*A	

A Bandelin Sonorex ultrasonic bath (model 1210; Walldorf, Germany) was used for degassing the solutions. Samples were incubated at 37 °C in a WB 22 (Memmert, Schwabach, Germany) or a TC-150 (Brookfield, Middleboro, USA) thermostat. For ultrafiltration experiments, a MPW-350R (JW Electronic, Warsaw, Poland) or a CM-50 M (ELMI Ltd., Riga, Latvia), operating at 10,000 and 1000–10,000 rpm, respectively, and different molecular mass cut-off filters (Amicon Ultracel; Millipore, Molsheim, France) were employed.

### Sample preparation

**Fig. 1:**
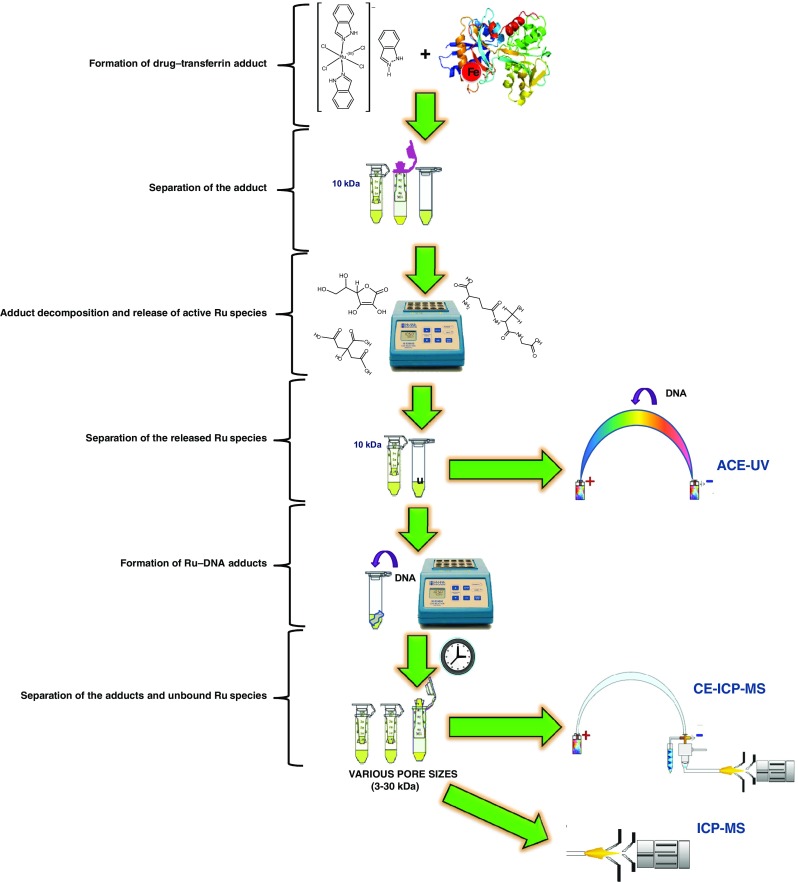
Analytical protocol for formation, isolation, and three-dimensional analysis of Ru–DNA species

A workflow template for sample handling is presented in Fig. 1. An aliquot of drug stock solution was mixed with 5 × 10^–5^ M solution of transferrin in 10 mM phosphate buffer, pH 7.4, containing 100 mM NaCl, to give a 2-fold molar excess of the drug over the protein in the final solution. The resultant mixture was incubated for 3 h at 37 °C to ensure complete adduct formation [18] and then ultrafiltrated through a 10 kDa cut-off filter for 30 min (37 °C) to isolate the adduct. After reverse ultrafiltration, the adduct solution (~30 *μ*L) was mixed with a solution mimicking cancer cell cytosol, to give a final adduct concentration of 5 × 10^–5^ M. The cytosol solution comprised 10 mM phosphate buffer (pH 6.0), 4 mM NaCl, glutathione (10 mM), ascorbic acid (10 mM), and citric acid (100 mM). The mixture was incubated for 24 h and then ultrafiltrated through a 10 kDa filter for 40 min. The ultrafiltrate was introduced into the capillary filled with 10 mM phosphate buffer (pH 6.0), containing 4 mM NaCl and 2 × 10^–7^–2 × 10^–5^ M DNA oligonucleotide (ACE), or mixed with the DNA oligonucleotide solution (final concentration of oligonucleotide 2 × 10^–6^ M). The mixture was incubated at 37 °C and aliquots were taken at a specified time (only after 24 h for ICP-MS) for ultrafiltration (40 min). A consecutive ultrafiltration with different combinations of filters was then performed. In the case of CE-ICP-MS, the initial ultrafiltrate was filtrated through a 30 kDa filter and the obtained ultrafiltrate through a 3 kDa filter, and both fractions (3–30 and <3 kDa) were analyzed. For off-line ICP-MS, the ultrafiltration sequence was a three-step in an order of 10, 5, and 3 kDa filter, following an appropriate dilution of Ru–DNA mixture with 2.5% nitric acid, so that the four fractions were subject to analysis.

## Results and discussion

### Intracellular activation of ruthenium drug

As a matter fact, among drug developers there is still no consensus regarding the mechanism of uptake and activation of the ruthenium drug of interest [10, 11]. According to the most often quoted concept of transportation, the drug enters the cell via transferrin route, being bound to this protein (though not the main binding partner in the bloodstream). The second popular hypothesis implies that within cancer cell the drug is activated to more reactive species, most likely by detaching transferrin and reduction to some Ru(II) species. A recent systematic study carried out in our laboratories focused on intracellular activation chemistry of a given Ru drug, including cancer cytosol environment [18–20]. Using a multidimensional metallomic approach, the evolution of a number of low molecular weight (MW) Ru species was ascertained, in which glutathione and ascorbic acid as the major bioreductants have a dominant impact on the drug–transferrin adduct. While the identification of ruthenium species bears so far a tentative character, this is not a constraint for the current investigation. Therefore, for consistency we have used here the same adduct formation and activation protocol as before (see Sample preparation section).

### Affinity CE

When affinity CE (ACE) is the method of choice, chemical processes comprising a metallodrug (as well as any other charged or under certain conditions, uncharged species) can be differentiated kinetically with regard to the time scale of a typical CE run (tens of minutes) [21, 22]. Rather fast reactions between the drug and a selected component of the background electrolyte (e.g., a bioligand) essentially attain the equilibrium during the time for which the drug stays in the capillary (more strictly speaking, is transported past the detector), giving rise to a binding response. With direct photometric detection, it can be displayed as two novel signals: a peak of the drug–bioligand adduct and a dip-peak attributable to a drop in the bioligand concentration (for a detailed view of all possible response scenarios, see ESM, Fig. S1). Otherwise, for fairly slow binding processes, one can expect no binding response (but only the free drug peak). Notably, such reactions are not discriminated against the CE assaying but passed to the realm of ordinary CE mode (using incubation of the reaction mixture for a certain period of time prior to analysis) [21].

It is important to point out that ACE analysis of chemical equilibria in metallodrug–bioligand systems has not yet become an accepted practice. Binding to proteins is known to proceed with comparatively slow kinetics (see [21] for a summary of kinetic data). For DNA, no published accounts on its interaction with metal-based drugs assessed by ACE can be traced in the literature. However, there are at least two reports by the group of Kane-Maguire and Wheeler [23, 24] indicative of that interactions between the metal complexes and DNA may be fast enough to exhibit a binding response in ACE. When DNA has been used in the electrophoretic buffer, resolution of the transition metal complexes into their optical isomers was observed. Such enantioselective effect can be explained by differences in isomer binding affinity toward DNA.

In order to simulate cancer cytosol electrolyte conditions, 10 mM phosphate buffer, pH 6.0, containing 4 mM NaCl, was chosen as a background electrolyte milieu. (We leave beyond the scope of this study the issue on the exact point where in-vivo interaction between active drug forms and DNA occurs, and cellular electrolyte settings at this point.) The DNA oligonucleotide concentration in the electrolyte was increased stepwise from 2 × 10^–7^ to 2 × 10^–5^ M. As the main parameter affecting analyte migration velocity and, inversely, the capillary residence (or reaction) time, the applied voltage was also subject to optimization. As a result of systematic variations in the range from 6 to 15 kV, a voltage of 10 kV, providing the optimal migration time and resolution, minimum peak broadening, acceptable running current, and also a lack of system peaks, was selected.

**Fig. 2:**
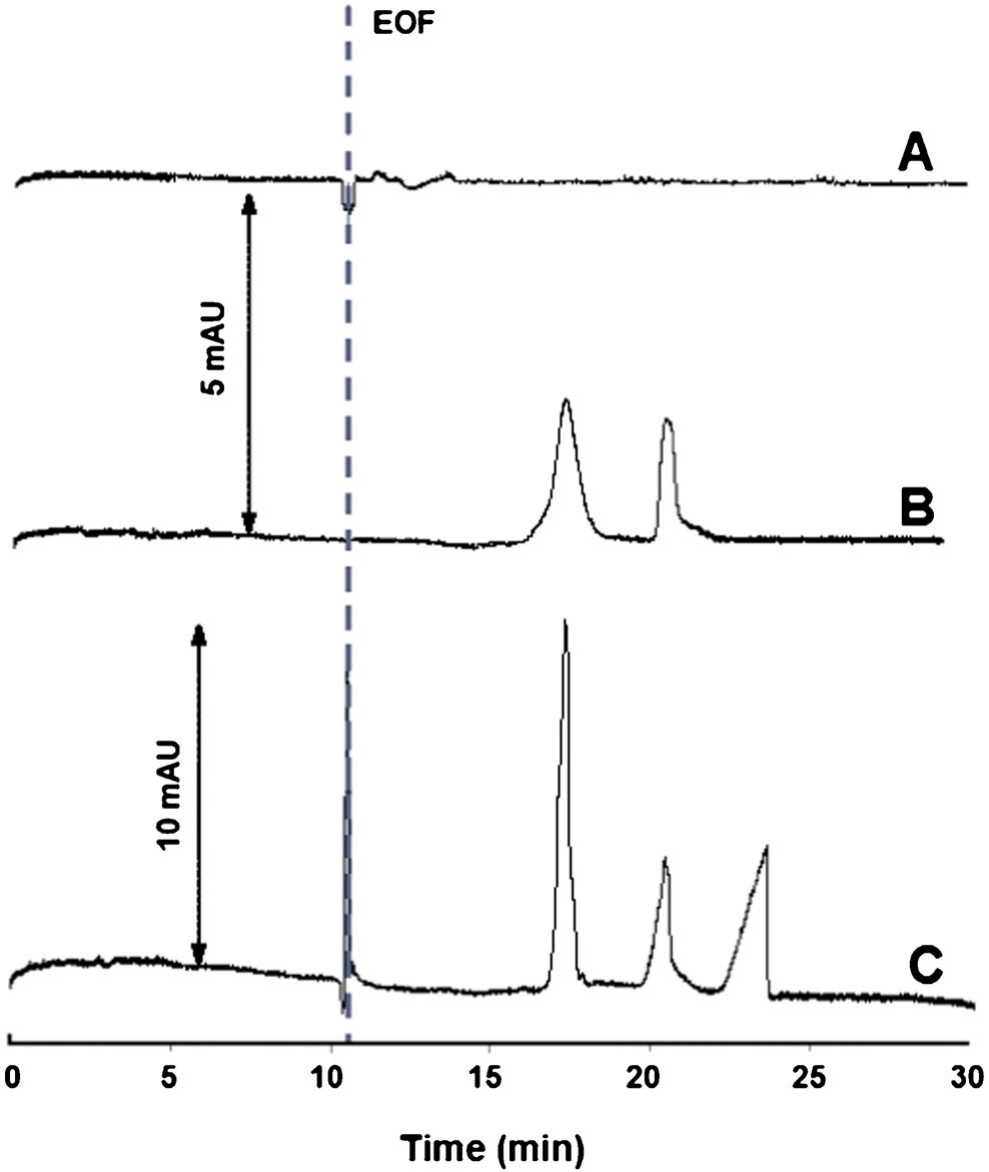
Electropherograms proving Ru–DNA binding. Sample: A – water (blank analysis); B, C – drug (in active form). Concentration of DNA in electrolyte: A, C – 2 × 10^–5^ M; B – zero. EOF = electroosmotic flow. Other ACE conditions, see Table 1

Upon injection of active drug forms (prepared as described above), there was no binding response at the lowest concentration of DNA, while certain changes were noticed to occur at 2 × 10^–6^ M; however, peak shape, intensity, and reproducibility were unsatisfactory. On the other hand, when using a 2 × 10^–5^ M DNA-containing electrolyte, the ACE system yielded a reproducible binding response (Fig. 2, trace C). It should be mentioned that without incorporating DNA, two well-resolved peaks of negatively charged Ru species were recorded (Fig. 2, trace B). This finding is in accord with our previous results [25], showing the occurrence of two active drug forms released from the transferrin adduct in the cytosol-like solution (see also CE-ICP-MS data below). Comparing traces B and C, it can be inferred that an additional peak, seen in trace C, is due to an DNA-binding product evolved in a ca. 20-min timespan.

### CE-ICP-MS

**Fig. 3:**
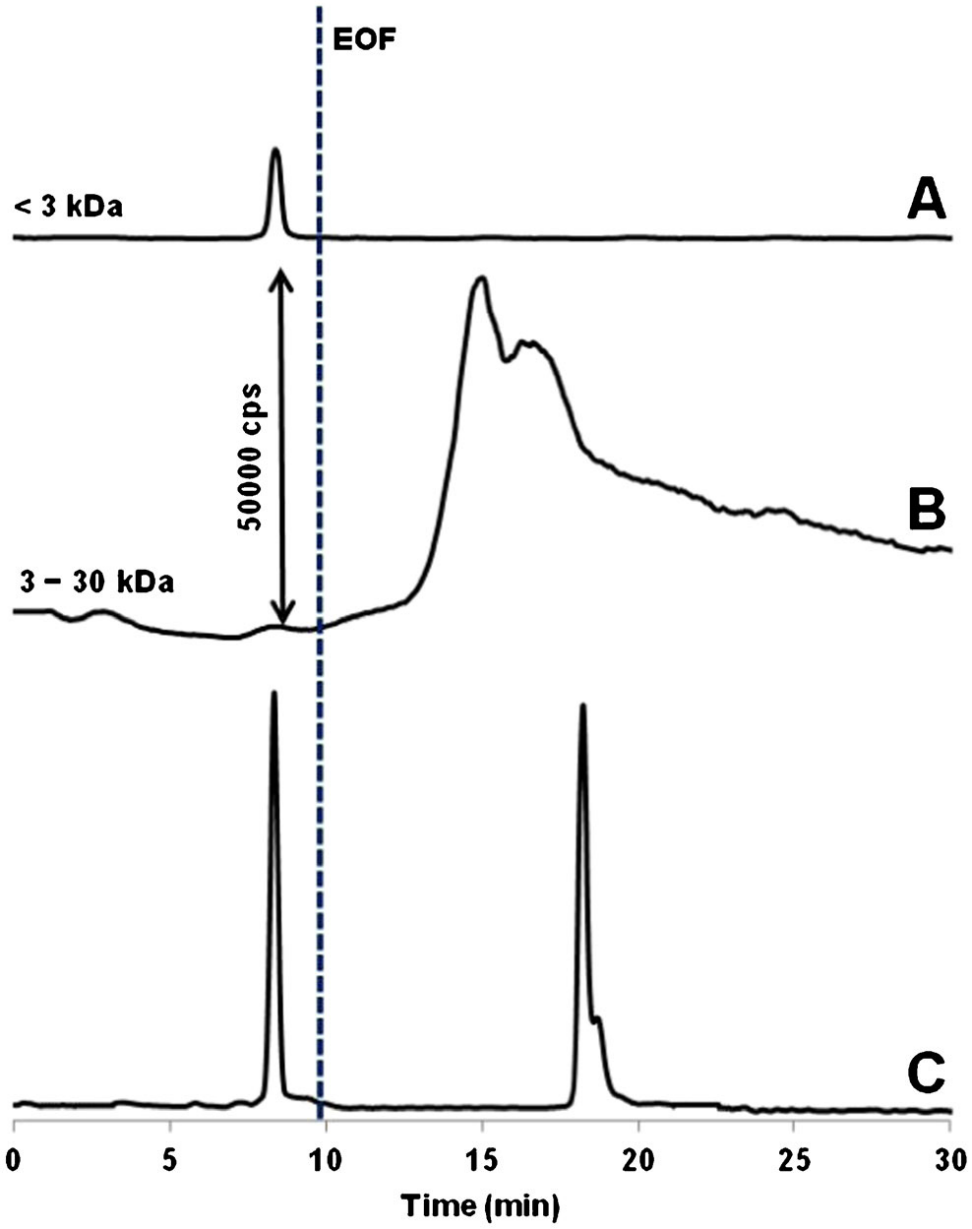
Various ruthenium species constituting drug’s active forms and originating from their 24-h interaction with DNA oligonucleotide. Trace C resulted from blank analysis (without oligonucleotide added). For CE and ICP-MS conditions, see Table 1

To gain further insight into the binding phenomenon, the active forms of the Ru drug were pre-incubated with oligonucleotide (up to 48 h) and subsequently analyzed by CE-ICP-MS (see experimental for ultrafiltration conditions). The same electrolyte buffer system was used in these trials as in ACE but void of oligonucleotide. The prerequisite of analyzing the two fractions as exemplified in Fig. 3, was that in fraction B (3–30 kDa) we expected to detect the signals of Ru–oligonucleotide adducts, while at <3 kDa in fraction A the low MW species of Ru could be monitored.

From Fig. 3 it is evident that at equilibrium conditions about 90% Ru are converted into high MW species (for comparative reasons, Fig. S2 in the ESM shows results of the CE-ICP-MS analysis of the same fractions after a shorter incubation time; note that one anionic form of Ru is still detectable). Apparently these species comprise at least two oligonucleotide adducts migrating as the overlapping peaks. It is important to note that in the studies on modeling physiological conditions, the selection of CE system parameters capable to enhance the resolution is typically restricted to very few variables, such as the applied voltage in our case. With regard to notable peak broadening, this was no great surprise as DNA oligonucleotides are known to adsorb onto the fused-silica capillary wall [24].

As shown in Fig. 3, even after prolonged incubation, a low MW species of Ru remains unattached to oligonucleotide and, importantly, migrates ahead of the EOF (i.e., in the migration range of positively charged analytes. Very likely the respective peak seen in trace A is due to [Ru^II^Hind(OH)(H_2_O)_3_]^+^ (Hind = 1*H*-indazole) identified as one of few (and the only cationic) ruthenium form released from the transferrin adduct under simulated cancer cytosol conditions [25] (cf., trace C). The fact that fraction A contains no negatively charged species can be inferred that non-hydrolyzed species, such as [Ru^II^HindCl_4_(GSH)]^2–^ and [Ru^II^HindCl_4_(GSSG)]^2–^ [25] (see two co-migrating peaks in trace C recorded in the absence of DNA), tend to interact more strongly with DNA.

### ICP-MS

It is useful to note that although CE-ICP-MS proved to be highly practicable as a speciation tool [16], the technique can be insufficiently sensitive when dealing with metal–bioligand species in real samples [14]. Even though this is not the case of the present study, we considered direct ICP-MS analysis to be offering viable supplementary information since through a series of ultrafiltration steps the content of the Ru species could fall beyond the limit of detection of CE-ICP-MS.

Analytical measurements of ruthenium originating from its drugs can be hampered by interfering *m*/*z* signals. A detailed investigation by Brouwers et al. showed, however, that neither ultrafiltrates, being free of proteins, nor the diluents, such as 1% nitric acid, but biological matrices cause a problem of interfering peaks at the mass-to-charge ratios of ruthenium [26]. Therefore, we have limited validation experiments on evaluation of unresolved spectral interferences to a blank (drug-free) analysis, as well as the analysis of DNA nucleotide as the only non-commercial chemical used. Both gave negligible interfering signals (≤4 cps). As an additional proof of no unanticipated spectral interferences, matching results were obtained by measuring ^101^Ru and ^102^Ru isotopes for all samples shown in Table 2.

**Table 2:** Results of the ICP-MS analysis (*n* = 3; *P* = 0.95)

Fraction	Concentration of Ru (×10^–6^ M)	Fraction content assignment
>10 kDa	1.0 ± 0.1	Adduct(s) with a Ru-to-DNA ratio of 1:2
5–10 kDa	7.2 ± 0.5	Mono-DNA adducts
3–5 kDa	<LOQ^a^	-
<3 kDa	1.3 ± 0.2	Free Ru

^a^LOQ: limit of quantification (1.2 × 10^–9^ M).

On the other hand, it was deemed obligatory to verify that all the sample preparation steps, particularly ultrafiltration, made no substantial analyte loss (e.g., associated with the sorption onto filter membrane or a plastic device). In the ultrafiltrate obtained after the treatment of the transferrin adduct with simulated cancer-cytosol solution, the Ru concentration was found within 10% of the nominal value (based on initial drug amount), indicating the satisfying recovery.

Table 2 gives a summary on the ruthenium distribution between fractions different in MW. As expected, the highest Ru level was found in the fraction bracketing the MW of DNA nucleotide (ca. 6 kDa). The amount of free ruthenium relative to the total amount of high MW Ru species conforms to the CE-ICP-MS data. What was less predictable, a rather high concentration of Ru exists in the fraction with the MW over 10 kDa. The most plausible explanation is that this is attributed to the formation of bis-DNA adducts.

## Conclusions

In summary, the present work demonstrates a proof of principle for investigating the interaction of metal-based drugs with biological targets. With the integrated use of CE and ICP-MS techniques, the formation of adducts between the experimental Ru(III) drug (importantly, as its active species to be possibly released in cancer cytosol) and DNA oligonucleotide has been confirmed. However, the binding system utilized to illustrate this platform is quite provisional, as the drug of choice is believed to primarily attack other biostructures inside the cancer cell (e.g., proteins like GRP-78), while the DNA model system selected takes into account only some elements of the *supposed in vivo* situation. Yet the binding information acquired suggests that the analytical approach described above may indeed be useful for assessing the intracellular fate of metallodrugs. Subsequent efforts to use this methodology will focus on different drug–biomolecule systems under circumstances matching as much as possible a real-world situation.

## Electronic supplementary material

Below is the link to the electronic supplementary material.

ESM 1 (PDF 213 kb)
