# Supplementary material for: Combination of ICP-MS, capillary electrophoresis, and their hyphenation for probing Ru(III) metallodrug–DNA interactions
Source: Anal Bioanal Chem. 2017 Jan 23;409(9):2421–7. doi: 10.1007/s00216-017-0186-0 (PMC5352744; doi:10.1007/s00216-017-0186-0)
Supplement: Supplementary file 1 — (PDF 213 kb) [file 216_2017_186_MOESM1_ESM.pdf]

Analytical and Bioanalytical Chemistry

Electronic Supplementary Material

Combination of ICP-MS, capillary electrophoresis, and their hyphenation for probing Ru(III) metallodrug–DNA interactions

Lidia S. Foteeva, Magdalena Matczuk, Katarzyna Pawlak, Svetlana S. Aleksenko, Sergey V. Nosenko, Vasily K. Karandashev, Maciej Jarosz, Andrei R. Timerbaev

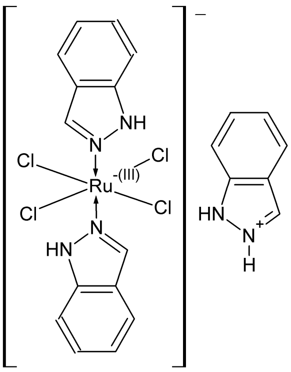

Scheme S1 Structural formula of the Ru drug

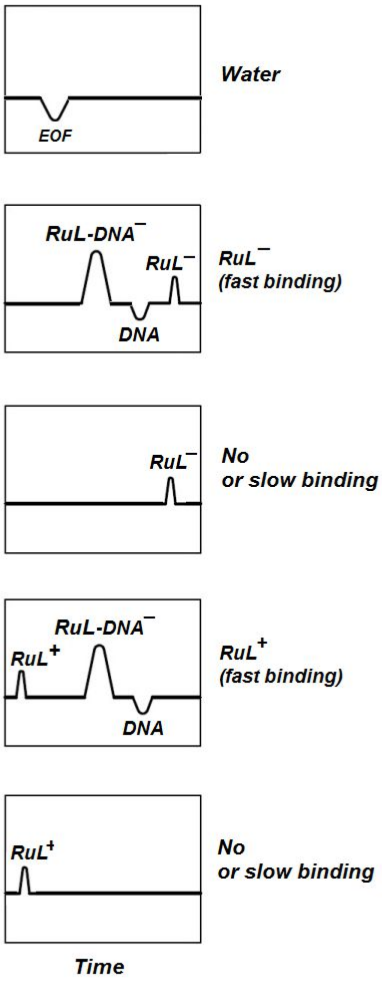

Fig. S1 Metallodrug–DNA binding situations differing in reaction rate and resultant theoretical ACE profiles (UV detection)

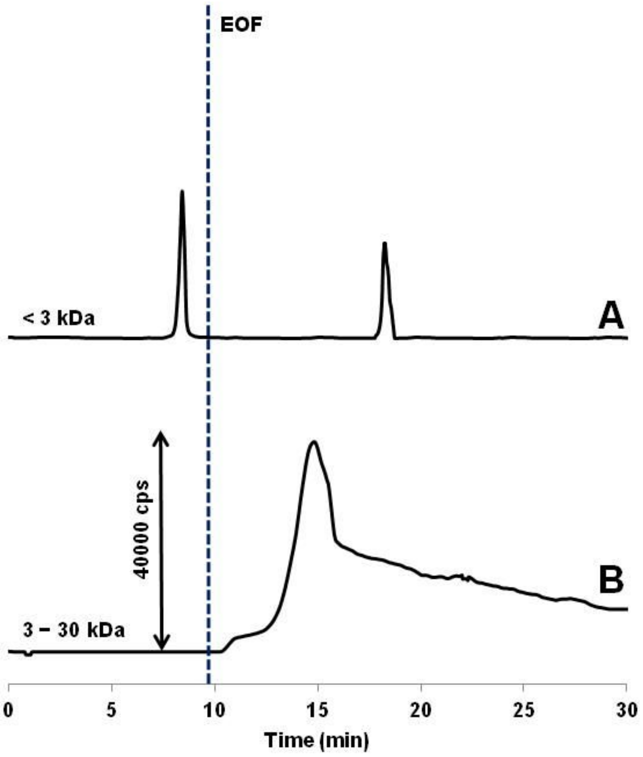

Fig. S2 Ruthenium speciation after 30 min of interaction with DNA oligonucleotide. For CE and ICP-MS conditions, see Table 1
